# The two chromosomes of the mitochondrial genome of a sugarcane cultivar: assembly and recombination analysis using long PacBio reads

**DOI:** 10.1038/srep31533

**Published:** 2016-08-17

**Authors:** Jeremy R. Shearman, Chutima Sonthirod, Chaiwat Naktang, Wirulda Pootakham, Thippawan Yoocha, Duangjai Sangsrakru, Nukoon Jomchai, Somvong Tragoonrung, Sithichoke Tangphatsornruang

**Affiliations:** 1 National Center for Genetic Engineering and Biotechnology, 113 Thailand Science Park, Paholyothin Road, Khlong Nueng, Khlong Luang, Pathumthani, 12120, Thailand

## Abstract

Sugarcane accounts for a large portion of the worlds sugar production. Modern commercial cultivars are complex hybrids of *S. officinarum* and several other *Saccharum* species. Historical records identify New Guinea as the origin of *S. officinarum* and that a small number of plants originating from there were used to generate all modern commercial cultivars. The mitochondrial genome can be a useful way to identify the maternal origin of commercial cultivars. We have used the PacBio RSII to sequence and assemble the mitochondrial genome of a South East Asian commercial cultivar, known as Khon Kaen 3. The long read length of this sequencing technology allowed for the mitochondrial genome to be assembled into two distinct circular chromosomes with all repeat sequences spanned by individual reads. Comparison of five commercial hybrids, two *S. officinarum* and one *S. spontaneum* to our assembly reveals no structural rearrangements between our assembly, the commercial hybrids and an *S. officinarum* from New Guinea. The *S. spontaneum*, from India, and one sample of *S. officinarum* (unknown origin) are substantially rearranged and have a large number of homozygous variants. This supports the record that *S. officinarum* plants from New Guinea are the maternal source of all modern commercial hybrids.

Sugarcane is the major source of processed sugar in the world and therefore an important crop species. Modern commercial cultivars of sugarcane are complex hybrids of *S. officinarum* and *S. spontaneum* and, to a lesser extent, some other species and hybrids (for review see^1^,^2^,^3^). Sugarcane is believed to have originated in the South Pacific, but was widely dispersed by early explorers making it difficult to pinpoint its origin. It is believed that *S. spontaneum* originates from India, but can be found growing wild from eastern and northern Africa, through the Middle East, to India, China, South East Asia, and through the Pacific to New Guinea. It is believed that *S. officinarum* have been derived from *S. robustum*, which shares the same center of origin with *S. officinarum* in New Guinea. Sequencing and comparing the nuclear and mitochondrial genomes may yield some insight into the history of sugarcane and provide a valuable resource for genetic improvement.

Plant mitochondrial genomes are remarkably different to animal mitochondrial genomes (for review see^4^). Plant mitochondrial genomes vary in size from 200 Kb in *Brassica hirta*^5^ to 11.3 Mb in *Silene conica*^6^. Genome expansion is primarily from repeat sequence, intron expansion and incorporation of plastid and nuclear DNA^7^,^8^. Accumulation of repetitive sequences in plant mitochondrial genomes cause frequent recombination events and dynamic genome rearrangements within a species leading to the generation of multiple circular DNA strands with overlapping sequence and different copy number^9^,^10^,^11^. In such cases the complete genome is referred to as the master circle with the DNA circles derived from recombination referred to as subgenomic circles or minicircles. It has been convention to represent the mitochondrial genome as a single DNA circle sometimes resulting in duplication of repeat sequence in the final assembly, however, this is not always noted^12^. In addition to this at least a few cases have been identified where the master circle no longer exists and the genome consists of multiple circular strands of DNA without shared sequence that could facilitate recombination^6^,^13^. Plant mitochondrial genomes are unlikely to be limited to a single origin of replication^14^,^15^. Break-induced repair and recombination has been proposed as a potential source for genome expansion and could be the cause for the long repeat sequences often found in plant mitochondria^16^. These long repeats plus DNA sequence sharing between the nuclear and plastid genomes can confound efforts to assemble plant mitochondrial genomes by introducing branch points that lead to multiple sequences including mitochondrial, nuclear or chloroplast sequence. This sequence sharing, the highly repetitive nature and relatively large size of plant mitochondrial genomes makes them difficult to assemble.

## Results and Discussion

### Sugarcane mitochondrial assembly

**Figure 1:**
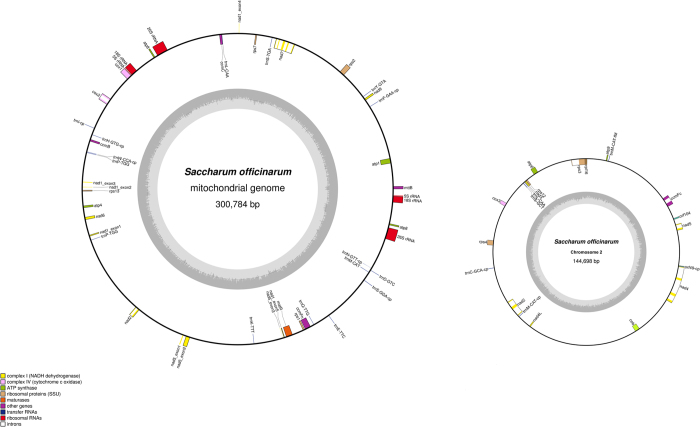
Chromosomes of the sugarcane mitochondrial genome. Chromosomes 1 and 2 of the sugarcane mitochondrial genome with gene location and symbol shown. Exons are shown in colour with small introns indicated as white space. Genes shown on the inside are on the negative strand while genes shown on the outside are on the positive strand. The grey circle represents the GC content.

An assembly from CAP3 using a subset of corrected reads >30 Kb consisted of 20 contigs which included 4 mitochondrial contigs and two chloroplast contigs with the remaining contigs coming from nuclear DNA based on blast results. The high number of nuclear contigs is a reflection of choosing a loose e-value blast cut-off so that all of the mitochondrial reads would be included to facilitate a complete final assembly. The four mitochondrial contigs could be joined to form two distinct circular chromosomes (Fig. 1) by using all of the corrected reads. The median read depth of all corrected reads to the two circular chromosomes was 13 and the mean read depth was 14. The largest chromosome, chromosome 1, is 300778 bp and includes a 15 Kb direct repeat sequence at 97558:113073 and 285262:300778 bp. There were reads that spanned both copies of the 15 Kb repeat sequence supporting that both copies occur in a single circular chromosome and no reads that supported any subgenomic circles from this sequence. The other chromosome, chromosome 2, is 144698 bp and forms a circular chromosome with no reads linking any sequence to chromosome 1.

While it is common for plant mitochondrial genomes to exist as a master circle with minicircles resulting from recombination between repeats, this is not the case for sugarcane. There were a total of 111 repeats in the mitochondrial assembly. The two largest repeats were the 15 Kb direct repeat and a 4 Kb inverted repeat. The remaining repeats were shorter than 360 bp with repeats in the range of 30–80 bp accounting for 87% of the total number of repeats. There were 47 repeats on chromosome 1, 11 repeats on chromosome 2 and 53 repeats shared between the two circular chromosomes. While the repeats shared between the two chromosomes could potentially facilitate recombination, the largest was only 296 bp so any recombination would have been easily detected by the long read lengths, yet none were found.

While no recombinations were found, a single alternate arrangement was identified for chromosome 1 that involves a 4 Kb inverted repeat that occurs at 45730–49805 bp and 169987–174062 bp with long reads spanning both copies. The alternate arrangement results in an inversion of the 120 Kb segment between the two repeats and deletion of one of the inverted repeats with five reads supporting the inversion versus seven reads supporting the arrangement we have presented. The lack of large repeats shared between the two chromosomes or sequences derived through recombination is solid evidence that the sugarcane mitochondrial genome exists not as a master circle with minicircles, but as two completely separate DNA circles. The mechanism by which plant mitochondrial genomes go from a combination of the master circle plus minicircles to multiple discrete DNA circles could be the break-induced repair and recombination events discussed by Christensen AC^16^.

### Sugarcane mitochondria annotation

We identified 66 unique open reading frames plus 26 duplicate copies and 17 partial chloroplast gene fragments (Table 1). These genes were primarily from the oxidative phosphorylation pathway (22 genes) and ribosome (10 genes). Fifty-seven genes are encoded by a single exon and eight genes are encoded across multiple exons. We found trans-splicing of group II introns in three genes: *nad1*, *nad2* and *nad5* (for review see^17^). The exons of *nad1* are separated by as much as 80 Kb and encoded on both the plus and minus strands of chromosome 1, consistent with findings in other species^17^. The genes *nad2* and *nad5* have exons split between chromosome 1 and chromosome 2, similar to what was found for *S. vulgaris*^6^. The RNA-seq data for six varieties plus genomic sequence for eight varieties from the DRA database was used to identify C → U RNA-editing in start and stop codons. Only *nad1* had confirmed RNA editing at a start codon, all of the database varieties had the base identified in our assembly (Cytosine) at this location and all of the RNA-seq varieties had a Uracil. No other cases of RNA-editing at start or stop codons were detected.

**Figure 2:**
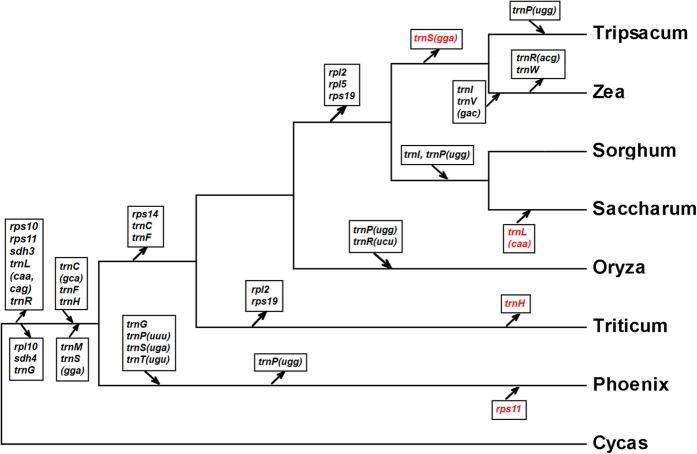
Phylogenetic tree comparing sugarcane with seven plant mitochondrial genomes. Gene gain and loss are indicated by arrow direction toward or away from the branch, respectively. Gene names in red indicate genes that have been lost and then regained or vice versa.

There were 18 tRNA genes identified, three of which occurred twice in the assembled mitochondrial genome (Table 1). Seven of the tRNA genes plus six other genes are from the sugarcane chloroplast (indicated with ‘-cp’ in Table 1) and primarily occur in the large sections of transferred chloroplast DNA. Gene transfer to and from the nucleus occurs commonly in mitochondria^18^. Sugarcane showed the same gene loss and gain as *Sorghum*, with one exception, sugarcane regained *trnL-CAA* (Fig. 2).

### Comparison with other species and sugarcane cultivars

**Figure 3:**
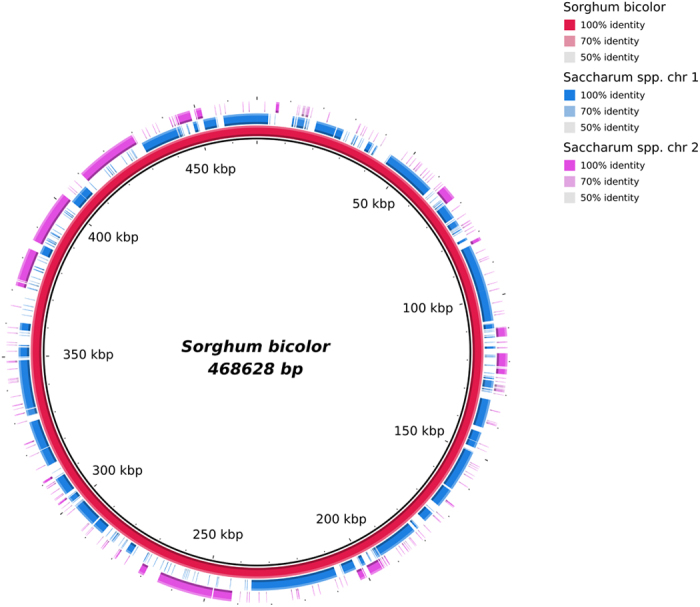
Comparison of sugarcane and *Sorghum bicolor* mitochondrial genomes. The *S. bicolor* genome is represented in full as the red circle. Similar sequence from the sugarcane assembly is represented in blue for chromosome 1 and purple for chromosome 2. The segmented nature of the two sugarcane chromosomes reflects the highly rearranged state of the sequence compared to *S. bicolor*.

We constructed a phylogenetic tree using 28 mitochondrial genes from seven species and found that sugarcane is most closely related to *Sorghum* (Fig. 2). The closest ancestor to sugarcane, of species with database sequence, has been identified as *Sorghum* by comparison of sugarcane BAC sequence^19^. The two species are close enough that Sorghum could be used as a template to assemble the majority of sugarcane genic DNA^19^. Blast against the mitochondrial genome of the closest species in the database, *Sorghum*, showed that 345 Kb of the 468 Kb mitochondrial genome is represented in our assembly, although, substantially rearranged (Fig. 3). This shows that a large amount of mitochondrial repeat sequence is shared between the two species. This includes 3 Kb of the inverted repeat and the entire direct repeat split into two parts, in both cases existing in the Sorghum genome as a single copy.

Database sequence from eight varieties, including one *S. spontaneum*, two *S. officinarum* and five hybrids, were used to identify variants. A large number of structural variants were identified between the *S. spontaneum*, *S. officinarum* and hybrids that we checked (Table 2). All of the structural variants found were in SES205A (*S. spontaneum*, accession: SRR922216) and sample 82–72 (*S. officinarum*, accession: SRR922217). The clone SES205A originates from India, but the origin of cultivar 82–72 could not be traced. Interestingly, the other sample of *S. officinarum*, IJ76–514, (accession: SRR528718), originally sourced from Irian Jaya (New Guinea), did not have the same structural variants, and instead was consistent with both our assembly and the other commercial hybrids. This is consistent with the hypothesis that all modern commercial varieties are derived from a New Guinean *S. officinarum*^3^. We performed *de novo* assemblies of the two samples with the structural variants (SES205A and 82–72) and the contigs from these assemblies supported the structural variants identified by the mapped reads, however, both samples had a large number of contigs which could not be constructed into complete genomes because of the high number of structural variants found. The most notable differences between these two samples and the others are multiple cases of reads joining Chromosome 1 with Chromosome 2. It is possible that these two samples have a single circular DNA strand instead of the two in our assembly or just a different arrangement involving two or more circles.

A total of 2,243 small variants were identified from the eight database samples consisting of 183 small InDels and 2,060 SNPs. We excluded any small variants with a per-sample minor allele proportion of less than 10% in an effort to exclude sequencing errors, which could not be reliably estimated from the database samples. The number of homozygous variants in most samples were small, in the range of 0 to 1%, with the exception of SES205A and 82–72, which both had 10%. This is consistent with the structural variations observed where these two samples were markedly different to the others. The majority of small variants identified (2,088) were shared by one to five samples (Table 3) and are therefore likely to have originated after sugarcane development. The remaining 155 variants were common to six or more samples and thus likely occurred early during sugarcane development.

## Conclusions

We have assembled the mitochondrial genome of a commercial sugarcane hybrid, Khon Kaen 3, and annotated coding sequence with the aid of RNA-seq data. Phylogenetic analysis supports the previous finding of *Sorghum* being the closest relative to sugarcane in the database. Although we only have two samples of *S. officinarum*, the similarity between the modern hybrid cultivars and IJ76–514 (SRR528718) is consistent with the hypothesis that *S. officinarum* from New Guinea was used to generate all modern commercial cultivars.

We have shown that the sugarcane mitochondrial genome exists as two discrete DNA circles with no evidence of recombination between them. However, the pronounced rearrangement between sugarcane and Sorghum shows that significant rearrangements have taken place in the past. The large number of sequences linking the two chromosomes in the sample of *S. spontaneum* and one of the samples (82–72) listed as *S. officinarum* show that the events leading to the separate chromosomes we identified here must have occurred relatively recently. This is consistent with divergence estimates from chloroplast sequence that show *S. officinarum* diverged from *S. spontaneum* between 580 and 780 thousand years ago^20^.

The large differences in size, structural arrangement and level of recombination between published mitochondrial genomes of different species suggest that plant mitochondrial genomes are in an interesting phase of evolution^11^. Sequencing additional species with long read length technologies is likely to yield additional insight to the evolution of plant mitochondrial genomes.

## Materials and Methods

### Sample and DNA extraction

The sugarcane sample is a commercial hybrid that has been developed in Thailand known as Khon Kaen 3. This cultivar was generated by crossing K84–200 (ROC1 x CP63–588) with 85-2-352 (SP70–1143 x Q76) and is a cultivar that is commonly used in South East Asia. Leaf tissue was collected from a single plant and used for DNA extraction with the standard CTAB method followed by clean-up using a DNeasy Mini spin column from Qiagen.

### Sequencing and assembly

DNA was used to prepare libraries for the PacBio RSII following the Pacific Biosciences ‘Procedure and Checklist −20 Kb Template Preparation Using BluePippin Size-Selection System’ protocol. DNA (10 ug) was sheared with a Covaris gTube, 4500 rpm for 2 minutes and the BluePippin cassette used was ‘0.75%DF Marker S1 high-pass 15–20 Kb’ with selection of 12–50 Kb. Sequencing was performed for 100 cells on the PacBio RSII. Raw reads longer than 26 Kb (118796 reads totalling 3.5 Gb) were used as seed reads and reads shorter than 26 Kb were used to correct them by the RS_PreAssembler.1 protocol with default settings from the Pacific Biosciences SMRTanalysis (v2.3.0) software package. The corrected reads were then blasted against the mitochondrial genome database from NCBI using an e-value cut-off of 1e–6 to identify reads that could be mitochondrial. A CAP3 assembly was performed using parameters: -o 1000 -e 200 -p 75 -k 0 with the corrected reads >30 Kb^21^. All the corrected reads were then blasted against the final assembly and contigs were joined that had overlapping sequence or reads that joined them to form a circular DNA strand. The corrected reads were mapped to this assembly using BWA MEM to confirm that the assembly was supported by the majority of reads and check for evidence of alternate genome configurations that could result from recombination^22^. Quiver (part of the SMRTanalysis suite) was then run on the final assembly to fix PacBio sequencing errors.

A data set of RNA-seq was obtained from DDBJ (SRR849062) for six pooled sugarcane cultivars and used to check for non-canonical start codons and RNA-editing^23^. Open Reading Frames (ORFs) were predicted using Open Reading Frame Finder [https://www.ncbi.nlm.nih.gov/gorf/gorf.html]. The tRNA genes were searched using tRNAscan-SE^24^. The annotated genes were also checked with the plant mitochondrial genome annotation program Mitofy^25^. All predicted ORFs, tRNA genes and rRNA genes were searched against the publicly available mitochondrial nucleotide and protein sequence database. Codon usage was calculated from all (33) of the mitochondrial coding genes. Repeats were identified using Reputer v3.0^26^.

### Sugarcane mitochondrial sequence comparison

A total of eight datasets of genomic sequence data from Illumina runs were downloaded from DDBJ^27^,^28^. These data sets included one *S. spontaneum* (SRR922216) sample, two *S. officinarum* (SRR922217 and SRR528718) and five samples listed as *Saccharum* hybrid (SRR528717, SRR871522, SRR922218, SRR922219, SRR922220). The reads from each data sets were mapped to the sugarcane mitochondrial assembly generated from this work using Bowtie2^29^, variants were called using GATK v3.4–46^30^ and structural variants were identified visually using IGV^31^. Small variants identified by GATK were only considered if the minor variant accounted for at least 10% of the reads on a per sample basis. Variants within repeat regions, including chloroplast sequence, were excluded. In addition, a de novo assembly using Ray^32^ was performed for two of the samples with the most structural variants (SRR922216 and SRR922217). The sugarcane mitochondrial genome was compared to the *Sorghum bicolor* mitochondrial genome using BLAST and graphed using the BLAST Ring Image Generator^33^.

### Phylogenetic tree

A phylogenetic tree was constructed using seven species (*Oryza sativa* Indica, *Phoenix dactylifera*, *Sorghum bicolor*, *Tripsacum dactyloides*, *Triticum aestivum*, *Zea mays* and *Cycas taitungensis* as an outgroup). Gene sequences from each species for 28 conserved genes (*nad1*, *nad2*, *nad3*, *nad4*, *nad4L*, *nad5*, *nad6*, *nad7*, *nad9*, *cob*, *cox1*, *cox2*, *cox3*, *matR*, *atp1*, *atp4*, *atp6*, *atp8*, *atp9*, *rps1*, *rps3*, *rps4*, *rps7*, *rps12*, *rps13*, *ccmB*, *ccmFN*, *mttB*) were compared and a maximum likelihood tree was constructed using MEGA 5 with 1000 bootstrap replications^34^. Gene gain and loss in sugarcane was determined by comparing the sugarcane gene content to database sequences of the other species used for the phylogenetic tree.

## Additional Information

**Accession codes:** Chromosome 1: LC107874 5674d093d25b2c19190110ce.Chromosome_1 Chromosome 2: LC107875 5674d093d25b2c19190110ce.Chromosome_2

**How to cite this article**: Shearman, J. R. *et al.* The two chromosomes of the mitochondrial genome of a sugarcane cultivar: assembly and recombination analysis using long PacBio reads. *Sci. Rep.* **6**, 31533; doi: 10.1038/srep31533 (2016).

## Figures and Tables

**Table 1:** Genes in the sugarcane mitochondrial genome.

Gene function	Gene name
Complex I	*nad1[5], nad2[5], nad3, nad4[4], nad4L, nad5[5], nad6, nad7[5], nad9*
Complex II	—
Complex III	*cob*
Complex IV	*cox1, cox2[2], cox3*
Complex V	*atp1, atp4, atp6, (2x)atp8, atp9*
Cytochrome-c biogenesis	*ccmB, ccmC, ccmFc[2], ccmFn*
SecY-independent transport	*mttB*
Ribosomal RNAs	*5S rRNA, 18S rRNA, 26S rRNA*
Ribosomal protein small subunit	*rps1, rps2, rps3[2], rps4, rps12, rps13*
Ribusomal protein large subunit	*rpl16*
Intron maturase	*matR*
Chloroplast transferred complete genes	*rpl14, rpl16, rpl23, rpl36, rps8, rps11*
Conserved Hypothetical genes	*orf25-cp, orf34-cp, orf74-cp, orf99-cp, orf104, orf137-cp, orf179-cp*
Transfer RNA	*trnC-GCA-cp, trnD-GUC, trnE-UUC, trnF-GAA-cp, trnH-GUG-cp, trnI-cp, trnK-UUU, (2x)trnL-CAA, (2x)trnM-CAU, trnM-CAU-cp, trnN-GUU-cp, (2x)trnP-UGG-cp, trnQ-UUG, trnS-GCT, trnS-GGA-cp, trnS-TGA, trnW-CCA-cp, trnY-GTA*
Pseudogenes	*sdh4*
cp-derived gene fragment transfer	*atpA, InfA, PetB, PetD, RpoA, atpB, atpE, atpH, ndhC, ndhJ, ndhK, orf251, rbcL, rpl2[2], rpoC1, rpoC2, rps19*

Bracketed numbers indicate copy number of each gene, square brackets indicate number of exons, chloroplast derived tRNAs have -cp appended to them.

**Table 2:** Structural variants.

Chr	Location (Kb)	Variant type	DRA accession and name							
			SRR922216, SES205A	SRR922217, 82–72	SRR528718, IJ76–514	SRR922218, B4362	SRR922219, RB72454	SRR922220, RB867515	SRR528717, Q165	SRR871522, SP70–1143
1	40.4	150 bp indel, 40.6 Kb (−) join 223.4 Kb (−)	1	1	0	0	0	0	0	0
1	45.7	120 Kb inversion	1	1	0	0	0	0	0	0
1	51.3–53.1	1.8 Kb deletion	1	1	0	0	0	0	0	0
1	82–82.5	500 bp deletion	1	1	0	0	0	0	0	0
1	83.5–98	Complex set of indels and rearrangements including links to chromosome 2	1	1	0	0	0	0	0	0
1	150.2–150.6	150.2 Kb (+) join Chromosome 2 5.4 Kb (+); 150.6 Kb (−) join Chromosome 2 5.5 Kb (−)	1	1	0	0	0	0	0	0
1	185–190	185 Kb (+) join 86 Kb (−); 190 Kb (+) join Chromosome 2 30 Kb (−)	1	1	0	0	0	0	0	0
1	196	196 Kb (+) join Chromosome 2 112 Kb (+)	1	1	0	0	0	0	0	0
1	196.9–197.3	400 bp deletiion	1	1	0	0	0	0	0	0
1	198–198.5	500 bp deletion	1	1	0	0	0	0	0	0
1	198.6–208.1	<10% average read depth suggesting nuclear DNA	1	1	0	0	0	0	0	0
1	222.8	222.8 Kb (+) join 235.6 Kb (+); 222.8 Kb (−) join 40.8 Kb (−)	1	1	0	0	0	0	0	0
1	235.6	235.6 Kb (+) join 222.8 Kb (+); 235.6 Kb (−) join 160.8 Kb (−)	1	1	0	0	0	0	0	0
1	262.7–263.2	500 bp deletion	1	1	0	0	0	0	0	0
2	29.4–30	29.4 Kb (+) join Chromosome 1 159.8 Kb (−); Almost zero read depth 29.4–29.9 Kb.	1	1	0	0	0	0	0	0
2	75.8–75.9	100 bp deletion	1	1	0	0	0	0	0	0
2	94.6	94.6 Kb (+/−) join Chromosome 1 120.8 Kb (+/−)	1	1	0	0	0	0	0	0
2	108.8	50 bp deletion	1	1	0	0	0	0	0	0
2	112.2	112.2 Kb (+) join Chromosome 1 195.7 Kb (+)	1	1	0	0	0	0	0	0
2	139–139.5	500 bp deletion	1	1	0	0	0	0	0	0

Presence of the variant is indicated by a 1 and a 0 represents the arrangement from our assembly.

**Table 3:** Small variant sharing between samples.

Number of Samples	Variant Count
1	823
2	774
3	259
4	140
5	92
6	63
7	43
8	33
9	12
10	2
11	2
